# How to assess haemodynamic impact of atrial fibrillation

**DOI:** 10.1093/ehjopen/oead123

**Published:** 2023-11-25

**Authors:** Naoya Kataoka, Teruhiko Imamura

**Affiliations:** Second Department of Internal Medicine, University of Toyama, 2630 Sugitani Toyama, Toyama 930-0194, Japan; Second Department of Internal Medicine, University of Toyama, 2630 Sugitani Toyama, Toyama 930-0194, Japan

**Keywords:** Arrhythmia, Tachycardia, Heart rate, Heart failure

**The authors response is available at https://doi.org/10.1093/ehjopen/oead126.**

The influence of atrial fibrillation on haemodynamics remains a subject of ongoing investigation. Almroth *et al*.^1^ have presented empirical evidence indicating that the induction of atrial fibrillation resulted in a discernible increase in diastolic blood pressure, a reduction in right ventricular end-diastolic pressure, and a concomitant decrease in right ventricular systolic pressure. A more extensive examination and deliberation are warranted to enhance the comprehensiveness of their finding.

It is imperative to note that the assessment of atrial fibrillation's haemodynamic repercussions was carried out within the context of general anaesthesia. The autonomic nervous system's influence, as well as systemic volume levels, can profoundly impact haemodynamics. General anaesthesia, owing to its denervation effect and peripheral vasodilation, effectively diminishes venous return. This can significantly perturb the haemodynamic equilibrium.

The heart rate is another pivotal factor with the potential to exert a substantial effect on haemodynamics. An augmented heart rate, for instance, may precipitate a decrease in venous return and compromise the right ventricular function.^2^ The authors reported an average increase of 43 beats per minute following atrial fibrillation induction.^1^ For a more rigorous comparative evaluation, it may be judicious to induce a corresponding heart rate elevation of 43 beats per minute in the control group through atrial pacing.

The authors gauged left atrial pressure through the intra-atrial septum employing the Brockenbrough method.^1^ It is pertinent to acknowledge that idiopathic atrial septal defects can impart a haemodynamic influence on bilateral atrial pressures. It is advisable to consider estimating left atrial pressure via pulmonary artery wedge pressure, employing right heart catheterization for a more accurate and refined assessment.

Lastly, it is imperative to recognize that the ramifications of acute haemodynamic changes may not persist into the later phases. Clinicians are often more apprehensive about the enduring haemodynamic effects of atrial fibrillation during the late phase. A haemodynamic evaluation conducted at the juncture when atrial fibrillation reverts to sinus rhythm may carry greater clinical relevance and significance in understanding the haemodynamic impact of atrial fibrillation on chronic phase.
